# Case report: A community case study of the human-animal bond in animal-assisted therapy: the experiences of psychiatric prisoners with therapy dogs

**DOI:** 10.3389/fpsyt.2023.1219305

**Published:** 2023-09-28

**Authors:** Sonia Smith, Colleen Anne Dell, Tim Claypool, Darlene Chalmers, Aliya Khalid

**Affiliations:** ^1^Department of Educational Psychology and Special Education, University of Saskatchewan, Saskatoon, SK, Canada; ^2^Department of Sociology, University of Saskatchewan, Saskatoon, SK, Canada; ^3^Faculty of Social Work, University of Regina, Regina, SK, Canada; ^4^Interdisciplinary Studies, College of Graduate and Postdoctoral Studies, University of Saskatchewan, Saskatoon, SK, Canada

**Keywords:** canine-assisted therapy, human-animal bond, Risk-Need-Responsivity model, therapy dog, attachment theory

## Abstract

Prisoners frequently experience chronic loneliness and lack social support, which can exacerbate their struggles with incarceration. According to attachment theory, individuals who are insecurely attached may be more likely to develop unstable relationships and engage in antisocial behavior as adults. In 2015 an animal-assisted therapy (AAT) program was implemented in a Canadian forensic psychiatric facility/prison, a “hybrid” facility that adheres to federal legislation regarding correctional services but follows provincial mental health legislation. The program centers on the development of a human-animal bond (HAB), which serves as a connection between the animals and prisoners. The HAB also addresses issues related to toxic masculinity, which are often present among men incarcerated in correctional facilities, including psychiatric prisons. An instrumental community case study design involving 6 prisoners at a forensic psychiatric facility/prison over 24 AAT sessions between 2015–2019 was undertaken. Interviews with the prisoners and their mental health clinicians were thematically analyzed to explore how the HAB was experienced as a form of attachment. Four themes emerged: safety, physical touch, reciprocity, and acceptance. These themes suggest that the therapy dogs have the potential to serve as a surrogate attachment figure for prisoners, mitigating their experiences of disconnection and fostering their development of interpersonal connections. This suggests attachment theory underpins the HAB and highlights the contribution of AAT practice and research in forensic psychiatric facilities/prisons. This study also suggests that the program's offering of prosocial support and nurturance/caring aligns with the specific criminogenic risks and needs identified within Correctional Service Canada's Risk-Need-Responsivity (RNR) model for rehabilitation. Continued research and attention should be paid to AAT programs as a valuable addition to the range of support networks available to prisoners in psychiatric or non-psychiatric institutions.

## Introduction

The literature surrounding the rehabilitation of prisoners emphasizes the significance of establishing caring interpersonal connections within correctional facilities, but in likelihood the conditions to do so are limited (1, 2). Individuals who are incarcerated often struggle to maintain connections with families and friends (3), leading to feelings of loneliness and isolation (4, 5). Although correctional facilities offer visitation, such visits can sometimes be problematic, including interpersonal reprimands (6), worsened dysregulated relationships (7), and can add to the stress and guilt experienced by those who are incarcerated (8). Further, geographical distance between an incarcerated individual and their home can make it even more difficult for them to access familial support systems (9).

Individuals who are incarcerated often seek support from staff members and fellow prisoners within correctional facilities to compensate for the lack of social connections outside the prison (3). However, pervasive mistrust and fear endemic to correctional institutions can make individuals who are incarcerated feel physically and emotionally insecure, hindering their ability to seek support (3). Moreover, factors such as congestion (10), power differences (11), and instances of violence and victimization (12), can exacerbate fearful feelings, further limiting individuals' capacity to form and maintain healthy social connections. This may be particularly challenging for those with psychiatric vulnerabilities who may already struggle with interpersonal difficulties, emotional dysregulation, and cognitive deficits. To cope with these challenges, prisoners may adopt unhealthy coping mechanisms that impede the formation of genuine and supportive social connections. For example, they may protect themselves by establishing a hypermasculine persona, masking their true emotions and presenting a hardened exterior (13), or form self-protective, aggressive bonds with others (14). Alternatively, they may avoid social interaction altogether, leading to isolation (14). These varied coping styles can undermine the formation of genuine and supportive social connections within a correctional facility, ultimately threatening the well-being of those incarcerated and their rehabilitation/treatment.

### Attachment theory

Attachment theory suggests that humans, in particular children, connect with other individuals who can provide them with physical and emotional support (15). This was first proposed by Bowlby in 1969. Ainsworth and colleagues built upon Bowlby's work (16) and suggested that a relationship between a caregiver and child can either be secure, insecure or a combination of both, depending on how well the caregiver can fulfill the needs of the child. Research has shown that children who have insecure attachment styles are more likely to exhibit a lack of empathy, remorse, and impulse control, as well as anger outbursts and interpersonal violence (17). These behaviors can persist into adulthood and contribute to antisocial behavior and incarceration (18, 19). Individuals who have an insecure attachment style and are incarcerated often find it difficult to form friendships and connections because they may be suspicious and distrustful (20). They may also struggle with establishing boundaries, feeling close to others, and expressing themselves (21).

The link between insecure attachment and criminal behavior may be worsened by mental illness (22). Prisoners with mental illness are more likely to have insecure attachment and perpetrate violence toward others (23). Forensic psychiatry recognizes attachment theory, but it is mostly used to identify criminal pathways rather than as a part of rehabilitation efforts. Research (24) shares that “attachment theory offers a framework against which clinicians can understand how individuals construct and deconstruct their world, and thus act upon the world in ways shaped by the emotional and cognitive images they hold of that world and the people in it, as well as mental representations of themselves and how they should behave” (p. 212–213). Others (25, 26) add that a better understanding of attachment and attachment styles can assist in guiding therapeutic approaches and mental health services within forensic psychiatry and other areas.

### Animals and attachment theory

Animals, particularly dogs, can provide important support and a sense of familial love for some individuals (27). Fine and Macintosh (28) describe this love as natural and instinctual. Burke et al. (26) recognize the social, psychological, biological, and neurological processes of attachment. The human-animal bond (HAB) is broadly defined by the American Veterinary Medical Association (29) as “a mutually beneficial and dynamic relationship between people and animals that is influenced by behaviors that are essential to the health and well-being of both. This includes, but is not limited to, emotional, psychological, and physical interactions of people, animals, and the environment” (online). Fine (30) notes that “the term human-animal bond was borrowed from the association found between parents and their offspring” (p. 6). Fine and Macintosh (28) suggest that the human-animal bond can be explained by three theories: attachment, biophilia and social support.

Barba (31) explains that the HAB emulates qualities similar to the attachment bond between a caregiver and a child, because pet owners care for and protect their companion animals who rely on them. The HAB can reflect characteristics of a secure attachment by promoting well-being and safety, and providing caregiving and commitment opportunities (32). Recent research suggests the HAB is like human-human attachment, with companion animals serving as a secure base (e.g., where comfort can be consistently sought) and a safe haven (e.g., comfort is sought in difficult times) for their owners (33). This has been observed recently in research focusing on the HAB specific to the COVID-19 pandemic (34, 35). Studies have also shown that owners strive to maintain a close relationship with their companion animals generally and experience distress when separated from them (36).

### Animal-assisted therapy

Over the past decade, rehabilitative programs in prisons involving animals, particularly dogs, have been introduced in Canada and other countries (37–42). Preliminary research on these programs in Canada has shown that they support participant rehabilitation by creating a positive social environment (43, 44), providing trustworthy communication opportunities, motivating participants to avoid disciplinary infractions and maintain pro-social behavior, and offering a sense of grounding (45) and social support through the HAB (46, 47). The newness of this programming and the lack of homogeneity amongst interventions makes evaluation across programs challenging (48). Therefore, it is crucial to empirically explore the HAB from the prisoners' perspective to understand their overall experiences, emotions, and thoughts as they interact with a dog in these programs (49, 50).

This community case study focuses on the application of animal-assisted therapy (AAT) as a form of attachment for prisoners in a Canadian forensic psychiatric facility/prison. AAT involves a licensed mental health professional working with volunteer therapy dog teams to carry out a measurable, controlled intervention that is goal-directed (30, 51, 52). The study is unique because it is the first of its kind known to the authors to consider the application of AAT as a surrogate or alternate form of attachment amongst a psychiatric prisoner population in Canada. This study builds upon existing research that has been conducted at the Regional Psychiatric Center (RPC) in the Canadian prairies. It examines how psychiatric prisoners experience the HAB with therapy dogs as a form of attachment in an AAT program.

## Context

RPC is one of five forensic psychiatric facilities/prisons in Canada. It serves as “hybrid” facility and adheres to federal legislation of the *Correctional and Conditional Release Act* but also follows provincial mental health legislation (53). It is a multi-level security institution that accommodates a diverse group of federal prisoners (i.e., those serving determinant custodial sentences >2-years-plus-a-day). It is mandated to accommodate prisoners “unable to function in parent institutions due to a mental disorder, cognitive impairment, and/or physical disability typically associated with aging, or who require specialized assessments”. Offenders must agree to be admitted to these facilities; otherwise, they must be certified by mental health legislation and/or mandated by the courts (54).

RPC offers an Animal Assisted Therapy (AAT) program in partnership with the St. John Ambulance Therapy Dog Program. From 2015 to 2019, correctional staff members at RPC identified about four participants per year for the AAT program based on factors such as mental health, limited social interaction, challenging substance use histories, and enjoyment of the company of a dog (55). The aim of the RPC AAT program is to offer psychiatric prisoners the opportunity to develop a HAB with therapy dogs through the dogs' perceived offering of comfort, love, and support in a goal-directed intervention. Preliminary evaluation of the program has found that participants experience a HAB involving unconditional love, acceptance, support, and the therapy dog being a trusted confidant (55). The program has also been identified to assist psychiatric prisoners in adhering to their correctional plan and regulating their emotions and affective states (55). However, it is still unclear if and how the HAB is experienced as a form of attachment, which is the focus of this case study.

### Sample

An instrumental community case study design was applied in this research. The study sample is comprised of 6 males, out of which four had an average of 20 years of incarceration while the remaining two had <20 years. The sample was all-male, which is consistent with previous research indicating that 94% of prisoners in Canada are male (56). Prisoners at RPC were referred to the AAT program by a correctional staff member for reasons denoted above (55). The AAT program exempted prisoners who staff questioned to pose a risk to the therapy dogs and handlers alike. This included prisoners with a history of violence toward animals. All participants in the study had positive interactions and experiences with dogs in the past.

Fulsome datasets are a challenge to gather at RPC due to frequent prisoner movement and program disruptions during crises. We have data for 6 prisoners over the time period. A total of 19 data sources from both AAT participants and RPC staff members were transcribed by a third party. There were 12 transcripts of participant interviews, which ranged from 48 to 10 min (with an average duration of 33 min), four staff member interview transcripts, which ranged from 57 to 37 min (with an average of 47 min), and three staff focus group transcripts, which averaged 1 h and 16 min. The duration of interviews varied dependent on the participants' ability to communicate their experiences and willingness to do so with the interviewers, who were also the therapy dog handlers. Interviews were conducted at the following times for participant one (at program completion, 8 months, and 3 years later), participant two (at program completion, and 4 months later), participant three (at program completion, and 4 months later), participant four (at program completion, and 9 months later), participant five (at program completion, and 10 months later) and participant six (at program completion). The program completion interviews were the most detailed, but data collection that occurred later on was also included because it offered important nuanced insight on the case study focus; that is, if and how forensic psychiatric prisoners experience connection with therapy dogs?

Interview questions for both prisoners and mental health staff aimed to understand the interaction between the participants and therapy dogs, including benefits of the connection, support, love, and comfort a participant might have experienced and how it was experienced. Key AAT participant interview questions included: “Are you generally glad to have met the therapy dogs?”, “Do you feel you have a connection with the therapy dogs? Explain.”, “Did you generally feel comforted/loved by the therapy dogs when you met him/her? ‘, “What does “comforted/loved” mean to you? Explain.”, “Did you feel generally supported by the therapy dogs when you met him/her?”, and “What does “supported” mean to you?”. Interview questions for mental health staff included: “Did you generally meet the goals of the sessions?”, “Were participants generally glad to meet with the therapy dogs? Why do you think this?”, “Do you think that the prisoners generally felt comforted/loved by the therapy dog? What does comforted/loved means to them in this context?”, and “Do you have any general observations about the prisoners' relationships with the therapy dogs applied in other areas at RPC?”.

### Participant details

AAT goals were established via the collaborative efforts of the participant, therapy dog teams, and the mental health staff who considered the participants' in-depth case history and mental health needs. Participants had unique mental health diagnoses including: Personality Disorders (General Personality Disorder, Antisocial Personality Disorder, Borderline Personality Disorder); challenging personality constellations (Antisocial, Borderline, Narcissistic, Dependent traits); Psychotic and related disorders (Schizophrenia, Schizoaffective Disorder), Substance Use Disorder and Alcohol Use Disorder; Neurodevelopmental Disorders (Intellectual Disability, Attention Deficit Hyperactivity Disorder); Major Depressive Disorder; and other neurological pathologies, including a history of seizures.

Participant 1 experienced physical trauma that left him self-conscious about his appearance and isolated from loved ones. He also had difficulty verbalizing his thoughts, feelings, and emotions due to mental health concerns. Participant 1's goals were to reduce his anxiety and stress levels and increase his self-esteem. Participant 2 had limited experience with dogs but recognized their potential benefits for his well-being. He experienced symptoms of anxiety and depression, struggled with anger, and had a small social network. The goal for him during the AAT program was to develop interpersonal trust. Participant 3 had participated in a dog training program at a previous prison, had mental health and addiction concerns, and difficulty trusting new people. He was compliant and passive and tended to avoid new experiences and individuals. His AAT goals aimed to help him develop trust, improve his social skills, and learn and practice mindfulness. Participant 4 had a substantial history of alcohol and substance use. He suffered from mental illness and physical health issues. He had a small social network on account of losing contact with his family and identified one person as his friend at RPC. His AAT goals included improving communication, increasing assertiveness, and expressing emotion. Participant 5 initially showed hesitation toward participating in the AAT program. His health concerns included mental health, low self-worth, and decreased emotional expression. His goals for the program included experiencing and expressing emotion, nurturing a positive sense of self, and practicing mindfulness. Participant 6 had previous positive experiences with dogs. He struggled with significant anxiety, self-criticism, interpersonal communication, and connection and was generally quite passive. The AAT goal for him was to increase pro-social assertiveness.

### Therapy dog team details

Dr. Colleen Dell, a professor and One Health and Wellness Research Chair at the University of Saskatchewan, and Dr. Darlene Chalmers, a registered social worker and associate professor of social work at the University of Regina, were the AAT dog handlers. Dr. Dell worked with three dogs: Subie, a 10-year-old Boxer; Kisbey, a 12-year-old Boxer; and Anna-Belle, an 8-year-old Bulldog (all ages at completion of data collection). Dr. Chalmers also worked with one of Dr. Dell's dogs, Subie, beginning in 2015. In 2017, Dr. Chalmer's two-year-old chocolate Labrador Retriever, Ruby, was introduced to the RPC program. All dogs and handlers passed the St. John Ambulance Therapy Dog program evaluation and met the program's requirements, including age, vaccination status and the dogs showed no behavioral problems (57).

### Mental health staff details

Each AAT participant was associated with a CSC staff member who was a registered mental health professional and a member of their core mental health team. The staff members co-facilitated the AAT sessions with the therapy dog team. Four female staff members participated in the AAT program: three social workers and one psychologist. The mental health staff members had varying lengths of experience in the correctional field and for each, this was their first exposure to AAT.

### AAT program

The AAT program consisted of 24 one-on-one sessions that took place weekly over 8 months. Twelve sessions were attended by Dr. Dell and 12 by Dr. Chalmers, with the four dogs in varying attendance. Each session lasted for 30 min, builds upon activities of the prior one, and began with a brief reintroduction to the therapy dog team to reinforce the HAB. The goal of each session was to help the participants achieve their individual goals that were established at the onset of the intervention, as detailed above. These included: practicing mindfulness; developing and practicing assertive communication skills; managing anxiety and depression; expressing emotions; and bolstering self-esteem. For instance, if the session goal was to improve assertiveness, the therapy dog could assist by performing simple training tasks with the participant, under the handler's direction, that would enable the participant to practice suitable body language and vocal tone. Alternatively, if a prisoner's goal was to experience emotional safety, the dog may have simply sat next to the prisoner for him to pet or hug.

### Data collection

Dr. Dell and Dr. Chalmers conducted semi-structured interviews with both the AAT participants and staff, and also facilitated staff focus groups. They made this decision because of the newness of the field and their practical experience with the program, which allowed them to ask especially insightful questions during the interview process. The questions were designed to understand the interaction between the participants and therapy dogs, as well as the benefits of the connection, support, love, and comfort a participant might have experienced and how it was experienced. In addition, Dell and Chalmers had established a rapport with both the program participants and mental health staff, which was beneficial in a psychiatric prison environment to conduct interviews. Their immersion in the lived experiences of the participants' interactions with therapy dogs helped them gain a deeper understanding of the data (58). Note that their observations, impressions, and insights were not a part of data collection.

### Data analysis

The study data was analyzed using thematic analysis, which involved categorically aggregating and interpreting the data to understand its significance (58). The study is oriented around the research question “Do psychiatric prisoners experience connection with therapy dogs, and if so, how?”. The aim of the thematic analysis was to explain the data by identifying meaning, categorization, and integration to provide an overall understanding (59). Themes were identified through comparison of all cases with one another and an ongoing attempt to understand the participants' perspectives. The process involved creating and refining themes until a final thematic framework was developed that represented the AAT program participants' experiences. To achieve this, the first author analyzed each transcript at least three times. Themes across multiple sources of transcripts were also compared to triangulate the data sources, which is critical for data analysis in case studies (58). Throughout the data analysis process, authors two and three provided reflection on the themes identified by the first author.

### Ethical considerations

The University of Saskatchewan Behavioral Ethics Review Board (Beh #1467) and Correctional Service Canada (CSC) (including RPC) approved the study. Approval for incorporating therapy dogs for therapeutic purposes in the study was provided by the Animal Research Ethics Board at the University of Saskatchewan (AUP 201330115).

## Details, results, and interpretation

Four interrelated themes emerged from the data analysis, offering insight on if and how prisoners in a Canadian psychiatric prison experience the HAB with therapy dogs. These themes were physical touch, safety, reciprocity, and acceptance. These themes, combined with existing knowledge from attachment theory and forensic psychiatry in non-carceral settings, suggest that the prisoners experienced the HAB as a form of attachment in the AAT program.

### Theme 1: physical touch

All participants in the AAT program reported connecting with the therapy dogs through physical touch, such as petting, hugging, cuddling, touching, and playing. Petting the therapy dogs was the favorite part of the program for most. One staff member described how “It's probably been two decades before he has had any contact with anybody, and then that really came up in those special moments. Just, kind of, laying and playing (with the dogs) which is really important to these guys because they don't even get touched.” The lack of comforting physical touch in most of the participants' lives while incarcerated is potentially similar to the absence they likely experienced in their early childhood. Connecting with the therapy dogs through physical touch may have helped fulfill the participants' need to feel safety and security, which Maslow (60, 61) posits as a basic human need. The therapy dogs appeared to provide a “secure base” for the participants, which is a key component of attachment theory. Given the insecure environment and lack of supportive, loving and reliable relationships that many psychiatric prisoners experience (62), encountering a safe haven has the potential to be particularly impactful (12, 63, 64).

Participants' and their mental health staff described that physical interaction with the therapy dogs fostered feelings of love, comfort, and support. This was especially evident for participants 1 and 6. This can be partially explained by research indicating that petting a dog can stimulate the release of oxytocin, the feel-good hormone, and reduce the production of cortisol, a stress hormone, in humans (65). A staff member described this for participant 1: “We can push him a little bit farther on personal stuff because he has that comfort level with Subie to do that, and Subie picks up on that. So, when we challenge, Subie gives him that physical comfort where he feels like he can get through it.” Similarly, for participant 6, a staff member shared: “when we just sat, and his anxiety was just able to be soothed by petting the dog, I found a huge difference”.

### Theme 2: safety

Participants' accounts, consistent with descriptions from mental health staff, suggested that they felt connected with the therapy dogs and emotionally and physically safe in their presence. This sense of safety seemingly helped them be more emotionally vulnerable in the AAT program. Participant 5 expressed this as: “I truly believe they got to see or feel who I really was as a person and I think that is why I got along with them so very well; is they knew that they were safe with me and I felt the same with them. You know I felt completely comfortable being in their presence, playing with them, I never felt anything negative when I was spending time with them.” This experience contrasted participants' likely experiences with and/or witnessing of violence in the past (12). The literature suggests that the human-animal attachment established through AAT is supported by experiences of safety and security (66), as demonstrated in this study. In a previous study, participants who were part of the AAT program at RPC also found the therapy dogs contributed to a safe environment for achieving their therapeutic goals (55).

Participants specifically reported that the connection with the therapy dogs allowed them to drop their hypermasculine persona or “mask” that they had to wear in prison to protect themselves in a hostile environment. This is a common challenge in a prison environment, as individuals adopt a defensive stance to safeguard themselves (12, 13, 63, 64). Participants 3 and 5 strongly expressed this sentiment. Participant 5 described his experience in the following way: “I am somebody with a good heart, has morals, positive beliefs and it is different from somebody like when I am back on the unit. Because, like I said, you have got to put this wall up and have to conduct yourself in a certain way because if you don't, people take advantage of you.” A staff member further explained that “He consistently spoke about how being in the program and with the dogs allowed him just to be genuine, to be real, to be raw, to shed whatever masks he feels he must wear sort of in the institution and to be himself…(He said) ‘you see a different piece or a different part of me', but he spoke about that here, too. Just not having to pretend, not having to act a certain way or have anything on show it was just all about being real and connecting and I think that allowed him a safe place to relax”.

### Theme 3: reciprocity

The participants in the study shared that they felt a strong connection with the therapy dogs due to the reciprocal nature of their interactions. They believed that the dogs reciprocated feelings of warmth, love, understanding, and support. Participant 5 said: “I felt that from all the dogs and I could see it and feel it in their eyes and how they interact with me, it was reciprocated. I have nothing but love and respect for them.” These reciprocal relationships were experienced and understood in three main ways.

The participants expressed feeling cared for by the therapy dogs and reciprocated this care toward them. For instance, participant 1 referred to the dogs as his family members and identified himself as the uncle of therapy dog, Subie. When asked to elaborate on this, he stated “because he (referring to himself) has no reason not to act that way”, given the love and care shown to him by the therapy dog. Participants also showed care for the therapy dogs by performing husbandry activities, such as giving them water or indicating when they needed to go outside to the washroom. These actions suggest a similarity between the human-animal attachment bond and caregiver-child attachment bond, where the animal relies on the owner for basic needs similar to a child (31). This congruence highlights the strong attachment and bond participants can feel toward the therapy dogs.

Second, mutual recognition played a role in participants' experiences of connection with the therapy dogs. They described how certain behaviors by the therapy dogs indicated that they recognized the participants, similar to how the participants acknowledged the dogs. Participant 1 described this mutual recognition as: “I definitely have a connection with them and you can see it as soon as I walk through that door, both of them (referring to Kibsey and Subie). Kisbey at first isn't really great, she stood (off) a little bit but as the weeks and months went by, you could start seeing her really taking to me; to the point that she is even crying (demonstrates the cry) just jumping on me, not tail wagging, body wagging.” Staff members also noted how participants frequently talked about the therapy dogs outside of AAT sessions and reflected on their mutual relationship with the therapy dogs during stressful situations at RPC. This continued connection between the participants and the therapy dogs demonstrates alternate attachment dynamics. Results from a study assessing the St. John Ambulance Therapy Dog program in correctional facilities support these findings, emphasizing how incarcerated individuals perceive empathy through reciprocal love and support (45, 55).

Third, many participants reported feeling a connection with the therapy dogs through what they perceived as empathy and mutual understanding. For example, participant 5 shared that he felt a connection with therapy dog Ruby because of the adversities she faced with a cancer diagnosis. He reflected, “I can relate to her. She has been through like a lot, having to go through (treatment) for having cancer.” He added “I have been through a lot myself. A lot of hardships.”. He highlighted how his connection grew after Ruby's leg was amputated. Mikulincer and Shaver (63) suggest that shared experiences, reciprocal support, trust, and self-esteem are all significant for attachment. Lalonde et al. (67) hypothesized that reciprocity nurtures sensations of affection, care, and comfort for therapy dog program participants—attributes consistent with secure attachment relationships (15). Therapy programs that incorporate animals have the potential to stimulate feelings consistent with secure attachment and provide consistent experiences of mutual respect and support, which may translate to other relationships for the participants (68).

### Theme 4: acceptance

Participants shared that they felt a strong connection with the therapy dogs because they believed the dogs accepted them unconditionally, with positive regard, and without judgment. This acceptance was reportedly experienced by all participants, and they felt that the dogs did not discriminate against them based on their histories, abilities, physical appearance, or goals during the AAT program. A staff member pointed out that participant 4 perceived a “lack of judgment” in the presence of the therapy dogs. Participant 1 shared that “they (the therapy dogs) made me feel special in a way that I was so very accepted, no ifs, ands or buts”. Research shows that individuals with mental illness who are incarcerated are at greater risk of experiencing humiliation and judgment due to the stigma of mental illness (69). However, studies on AAT in Canadian prisons have found that therapy dogs can provide complete acceptance to individuals who are incarcerated (9, 45, 55, 70). Chandler (71, 72) shares how dogs can provide authentic and consistent positive regard to participants in therapeutic interventions due to being perceived as non-judgmental and bias-free. This is reflective of the warmth and acceptance that can be provided by a secure attachment figure (63, 73). Previous evaluations of prison AAT programs have also noted that the perception of dogs as providing unconditional positive regard can improve the self-esteem of participants (74, 75).

This study's results suggested that acceptance plays a key role in happiness. A staff member mentioned that “happy here does not mean the same as happy in the community” but rather “those little moments where you feel truly accepted and free to be who you are.” The participants experienced happiness in different ways, but they all shared the feeling of acceptance. Participant 3 said: “they (the therapy dogs) make me feel very happy; they make me feel very wanted, alive for one half hour in there. It is almost euphoric, right?”. Previous research has shown that dogs can boost morale and happiness in correctional facilities (74). These findings provide further evidence for the attributes of human-animal attachments and bonds, as highlighted by Hazan and Shaver's (73) identification of the significance of care, trust, and esteem in establishing a connection. This supports the recognition of AAT as providing a secure base to participants, with therapy dogs serving as substitute empathic connections for various rehabilitative practices (76).

## Discussion

Thomas and Matusitz (76) suggest that the HAB between a prisoner and therapy dog can serve as an alternative empathic relationship to support prisoner rehabilitation. This is in line with attachment theory, which explains that prisoners can develop a bond with therapy dogs based on trust and unconditional love, and the dogs can act as a substitute attachment figure (77). Sable (32) also notes similarities between human-animal attachment and human-human attachment in terms of feelings of safety and security. This is likewise supported in the work of Fine (27). This study identified four themes (safety, physical touch, reciprocity, acceptance) that describe how a HAB is developed for AAT participants in a psychiatric facility/prison. The study highlights that therapy dogs can serve as a secure and surrogate attachment figure for AAT participants, mitigating their experiences of disconnection and fostering their development of interpersonal connections. This suggests that the program's offering of prosocial support and nurturance/caring align with specific criminogenic risks and needs identified within Correctional Service of Canada's (CSC) Risk-Need-Responsivity (RNR) model for rehabilitation.

The Correctional Service of Canada oversees programming and assessment in federal corrections, guided by the RNR model (78). The RNR model aims to rehabilitate incarcerated individuals and reduce recidivism by providing evidence-based treatment that considers individuals' risk levels and criminogenic needs (79, 80). This model applies equally to CSC's prisons and forensic psychiatric facilities/prisons. Prisoners at the forensic psychiatric facility where this study was conducted (i.e., RPC) have limited ongoing support systems, lengthy period of incarceration and/or isolation from others and varied insecure attachment style behavioral patterns that hinder the development of an adequate social support system. Therapy dogs helped prisoners feel safe, warm, cared for, and supported, possibly fulfilling the criminogenic need of “familial/marital relationships” within the RNR model (80). They also helped prisoners develop pro-social contact, countering the “social supports for criminal risk” associated with the RNR model (80). Overall, the alternate attachment relationship between prisoners and therapy dogs reinforces the therapeutic process within AAT by alleviating social disconnection and fostering the growth of new interpersonal relationships within the participants' support network (76).

As we and other researchers continue to explore this new area, it will be important to conduct more robust studies that consider the unique lived experiences and needs of incarcerated women. Female prisoners have been shown to have greater familial and marital needs and a higher risk of interpersonal victimization (81). We must also consider non-binary individuals as participants in these studies. Second, it is essential to include participants who identify as Indigenous (two did in this study), as they make up a significant proportion of the Canadian federal prison population (82). To do so would be in alignment with Canada's commitment to decolonization, including in the prison context (83). The Truth and Reconciliation Commission Calls to Action emphasize the need to implement and evaluate community sanctions (84), which should include connection to animals as part of a holistic approach to wellness (85). Third, it is essential to acknowledge the importance of the therapy dog handlers, who work in tandem with the dogs, and the role of mental health staff who lead the AAT sessions. Neglecting to consider their impact limits the findings of this study. The value of doing so, for example, is identified in the work of Andrews et al. (79), who highlight the significance of a therapeutic alliance between individuals who are incarcerated and intervention providers when considering the RNR model responsivity principle.

With CSC building a new forensic facility in Atlantic Canada, it presents an opportunity to consider AAT and other initiatives that involve animals. AAT is one such intervention, but there are other ways to incorporate animals, such as providing outdoor spaces/courtyards to appreciate animals in their natural habitat (e.g., watching wild birds) or offering animal-related programs, like the Dog House program at Fraser Valley Institution. This program is an employment training program for prisoners that focuses on handling, grooming, and training dogs. CSC could also consider allowing prisoners to visit with their own pets; the importance of the human-animal bond in CSC facilities was relayed in a 2019 project titled Animal Memories (86). CSC's Commissioner Anne Kelly acknowledged the importance of animals in people's lives in the project magazine's forward.

Overall, according to Rich (24), attachment theory plays a crucial role within forensic psychiatry

as a tool that can help us recognize how connections are made, how they are damaged and how they took shape in each individual with whom we work. Whether in the forensic or the general mental health setting, an attachment-informed framework helps us to see and understand our patients better, and recognize how to form, re-form and re-activate a sense of being understood, and thus become more attached to others (p. 215).

Therapy dogs, and animals generally, are an overlooked source for human of attachment in forensic facilities.

## Future considerations

To begin exploring the role of attachment between humans and animals in forensic psychiatry, one potential starting point is for professionals to enroll in the Therapy Dog handler course, for which there is no cost. This course provides valuable insight into the role of therapy dogs in different environments, including forensic facilities/prisons. Interested individuals can visit https://sites.usask.ca/online-handler-education/ for more information. If there is interest, the authors on this paper can look at acquiring continuing education hours through the Royal College Maintenance of Certification program in Canada for completion of the 8-hour course.

## Data availability statement

The datasets presented in this article are not readily available to safeguard prisoner privacy. Requests to access the datasets should be directed to SS via email, sonianatsmith@gmail.com.

## Ethics statement

The studies involving humans were approved by University of Saskatchewan Behavioural Ethics Review Board (Beh #1467). The participants provided their written informed consent to participate in this study. The animal studies were approved by University of Saskatchewan Animal Research Ethics Board (AUP 201330115). The studies were conducted in accordance with the local legislation and institutional requirements. Written informed consent was obtained from the owners for the participation of their animals in this study. Written informed consent was obtained from the individual(s) for the publication of any potentially identifiable images or data included in this article.

## Author contributions

SS contributed to conceptualization of the research topic, data analysis, and data interpretation. CD and DC contributed to data collection. SS prepared the draft of this paper, which was reviewed, input included, and approved by CD, TC, DC, and AK. AK contributed to preparing the article for publication which was reviewed, revised as needed, and approved for submission by CD, TC, DC, and SS. All authors agree to be accountable for the article in its entirety as it pertains to the integrity and/or accuracy of work. All authors contributed to the article and approved the submitted version.
