# Supplementary material for: Case report: A community case study of the human-animal bond in animal-assisted therapy: the experiences of psychiatric prisoners with therapy dogs
Source: Front Psychiatry. 2023 Sep 28;14:1219305. doi: 10.3389/fpsyt.2023.1219305 (PMC10568475; doi:10.3389/fpsyt.2023.1219305)
Supplement: Supplementary file 1 [file Data_Sheet_1.PDF]

Theme	Participants' Responses	Staff Members' Responses
Physical Touch		It's probably been two decades before he has had any contact with anybody, and then that really came up in those special moments. Just, kind of, laying and playing [with the dogs] which is really important to these guys because they don't even get touched.<br><br>*For Participant 6*<br>When we just sat, and his anxiety was just able to be soothed by petting the dog, I found a huge difference.
Safety	*Participant 5*<br>I truly believe they got to see or feel who I really was as a person and I think that is why I got along with them so very well; is they knew that they were safe with me and I felt the same with them. You know I felt completely comfortable being in their presence, playing with them, I never felt anything negative when I was spending time with them.	*For Participant 3*<br>If you don't have a safe place to be yourself, if you don't have a safe place to actually learn who you are. Because, I think in this environment, when they wear masks so often and sometimes 24/7, I think anyone of us after how many years he [referring to participant] has been in would lose sight as to who am I really and what do I really value.
	Participant 5<br>I am somebody with a good heart, has morals, positive beliefs and it is different from somebody like when I am back on the unit. Because, like I said, you have got to put this wall up and have to conduct yourself in a certain way because if you don't, people take advantage of you.	*For Participant 5*<br>He consistently spoke about how being in the program and with the dogs allowed him just to be genuine, to be real, to be raw, to shed whatever masks he feels he must wear sort of in the institution and to be himself…[He said] 'you see a different piece or a different part of me', but he spoke about that here, too. Just not having to pretend, not having to act a certain way or have anything on show it was just all about being real and connecting and I think that allowed him a safe place to relax.

Reciprocity	*Participant 2*<br>they are happy to see me and get [to be] around me, too, because I am also happy to be around them too…I was nice to them and they are nice to me.<br><br>*Participant 1*<br>I definitely have a connection with them and you can see it as soon as I walk through that door, both of them (referring to Kibsey and Subie). Kisbey at first isn't really great, she stood [off] a little bit but as the weeks and months went by, you could start seeing her really taking to me; to the point that she is even crying [demonstrates the cry] just jumping on me, not tail wagging, body wagging.	They (the participants) just feel such a connection with the dogs and the dogs are so good. The dogs, you know, are just genuinely enjoying being there; you are not sitting back and saying to yourself "well those dogs are just being nice because I have a treat in my hand", they want to be there.
Acceptance	*Participant 1*<br>They [the therapy dogs] made me feel special in a way that I was so very accepted, no ifs, ands or buts.<br><br>*Participant 4*<br>They (the therapy dogs) have always given me like this positive energy and must make me feel good about myself being in their presence, and also when I left.<br><br>*Participant 3*<br>They [the therapy dogs] make me feel very happy; they make me feel very wanted, alive for one half hour in there. It is almost euphoric, right?	Happy here does not mean the same as happy in the community.
